# Reproducibility of phase-contrast MRI in the coronary artery: towards noninvasive pressure gradient measurement and quantification of fractional flow reserve

**DOI:** 10.1186/1532-429X-17-S1-Q11

**Published:** 2015-02-03

**Authors:** Zixin Deng, Qi Yang, Xiaoming Bi, Zhaoyang Fan, Debiao Li

**Affiliations:** 1 Cedars Sinai Medical Center, Los Angeles, CA, USA; 2 Bioengineering, University of California, Los Angeles, Los Angeles, CA, USA; 3 R&D, Siemens Healthcare, Los Angeles, CA, USA

## Background

Fractional Flow Reserve (FFR) is an invasively determined index of the functional severity of an intermediate coronary stenosis by measuring the pressure drop across the lesion [1]. Noninvasive pressure gradient (ΔP) measurements using phase-contrast (PC)-MRI have been attempted in the aorta, carotid, and renal arteries [2-4]. The purpose of this study is to assess the reproducibility of PC-MRI and noninvasive ΔP calculations in the coronary artery, which is relevant for establishing the robustness of the noninvasive FFR technique.

## Methods

2D PC-MRI was used to acquire two-cardiac-phase data at mid-diastole and end-expiration via ECG-triggering and navigator-gating on 3T MAGNETOM Verio (Siemens). K-space phase-encoding ordering is designed to allow offline view sharing [5], which is applied in cases where the acquisition window exceeds the quiescent period (~100ms). The sequence measures the velocity field (v_x_, v_y_, v_z_) of a single cross-section per acquisition and 4-5 consecutive slices were obtained in the proximal LAD. Reproducibility was assessed with two repeat scans on 4 healthy subjects. VENC ranged 30-45 cm/s for each flow encoding direction was determined from a VENC scout scan. The Navier-Stokes equations were used to derive ΔP [6]. In addition, a flow phantom (gadolinium-doped water flow at 300 mL/min in a silicone tubing of 4.8mm ID) with 40% stenosis (VENC=130z30xy cm/s) was likewise tested for reproducibility. Imaging parameters were: in-plane resolution = 0.58-0.67mm, slice thickness = 3.2 mm, flip angle = 15°, 65-71 ms/phase with the first phase strictly coinciding with the quiescent period, scan time = 1-3 min per slice. Absolute maximum and averaged velocities at each slice in all three directions and the ΔP between adjacent slices obtained from both scans were statistically compared via intra-class correlation (ICC).

## Results

Volunteer studies: averaged maximum through-plane velocity over all healthy volunteers was 16.5±4.0 cm/s. A total of 19 slices were acquired from all subjects. For velocity measurements, excellent correlations were seen in the through-plane velocities (v_z_), with ICCs of 0.93/0.96 and slightly lower in v_x_ and v_y_ with ICCs of 0.83/0.86 and 0.80/0.78 for cardiac phases 1 and 2, respectively. For ΔPs, ICC was 0.51 with an average of 0.1039±0.28 mmHg among all subjects. Phantom studies: stenosis with 40% narrowing showed excellent correlations in all velocity directions and ΔPs (table 1).

**Table 1:** Intra-Class Correlation (ICC) between two scans.

	Velocity Encoding Direction	Averaged Velocity		Absolute Maximum Velocity		Pressure Gradient (ΔP)
		Phase 1	Phase 2	Phase 1	Phase 2	r = 0.508 p<0.05
	
Volunteers	Z	0.932	0.968	0.935	0.959	
		
	X	0.578	0.447	0.828	0.861	
		
	Y	0.931	0.918	0.804	0.779	

Phantom	Z	0.992		0.988		r = 0.768 p<0.05
		
	X	0.918		0.934		
		
	Y	0.979		0.969		

## Conclusions

Our preliminary results suggest that the noninvasive quantification of flow velocities and ΔPs are reproducible in the coronary arteries, demonstrating the robustness and feasibility of 2D PC-MRI. Patient studies are underway to determine ΔP and FFR thresholds between healthy and patient populations. Further technical improvements are warranted to reduce noise and improve reproducibility.

## Funding

N/A.
